# Acute graft rejection mimicking constrictive pericarditis after heart transplantation. A case report

**DOI:** 10.47487/apcyccv.v5i2.339

**Published:** 2024-06-24

**Authors:** Lucrecia María Burgos, Franco Nicolás Ballari, Rocío Consuelo Baro Vila, María Antonella de Bortoli, Mariano Vrancic, Mirta Diez

**Affiliations:** 1 Instituto Cardiovascular, Ciudad Autónoma de Buenos Aires, Buenos Aires, Argentina. Instituto Cardiovascular Ciudad Autónoma de Buenos Aires, Buenos Aires Argentina

**Keywords:** Heart Transplantation, Graft Rejection, Pericarditis, Constrictive, Trasplante de Corazón, Rechazo del Injerto, Pericarditis Constrictiva

## Abstract

Constrictive pericarditis (CP) is an infrequent complication following heart transplantation (HTx) and arises from diverse postoperative occurrences, including mediastinitis, pericardial effusion, or allograft rejection. Indeed, this rare clinical entity can be misdiagnosed as a rejection episode or restrictive cardiomyopathy. In this report, we present the case of a 43-year-old male who underwent HTx 1.5 years prior and was subsequently admitted to our center due to the gradual onset of symptoms indicative of right congestive heart failure, with an initial diagnosis of constrictive pericarditis.

## RESUMEN

La pericarditis constrictiva (PC) representa una complicación rara después de un trasplante de corazón (TC), derivada de diversos eventos posoperatorios como mediastinitis, derrame pericárdico o rechazo del injerto. De hecho, esta entidad clínica poco común puede ser diagnosticada erróneamente como un episodio de rechazo o miocardiopatía restrictiva. En este informe presentamos el caso de un hombre de 43 años que se sometió a un TC 1,5 años antes y que fue ingresado posteriormente a nuestra institución debido al inicio gradual de síntomas indicativos de insuficiencia cardíaca congestiva derecha, con diagnóstico inicial de pericarditis constrictiva.

## Introduction

Constrictive pericarditis (CP) stands as a rare, yet noteworthy complication in the post-cardiac transplant population, manifesting clinically as progressive heart failure ^1^. Defined as an inflammatory condition, CP is characterized by the encasement of the heart within a rigid, non-pliable pericardium, resulting in restricted diastolic filling. The multifactorial etiology of CP in heart transplant recipients encompasses diverse postoperative events, including mediastinitis, pericardial effusion, and allograft rejection ^2^.

Here we describe a case of apparent CP in a post cardiac transplant patient without obvious risk factors. 

## Case report

We present the case of a 43-year-old man with idiopathic dilated cardiomyopathy who underwent bicaval orthotopic heart transplantation (HTx). Postoperative complications included moderate pericardial effusion. He received a triple-drug immunosuppressive regimen with oral corticosteroids (Prednisone), a purine synthesis inhibitor (Mycophenolate mofetil), and a calcineurin inhibitor (Tacrolimus). Subsequent controls showed preserved graft function, without rejection in elective endomyocardial biopsies (EMB) or relevant clinical events at follow-up.

**Figure 1:**
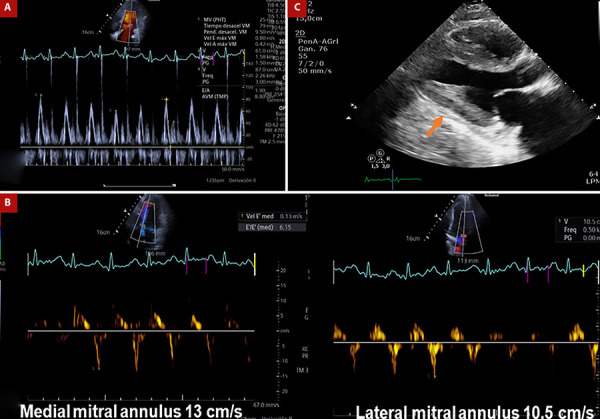
(A) Pulsed-wave Doppler spectrum of mitral inflow velocities demonstrates a marked respiratory variation of the peak E-wave velocity with short deceleration time. (B) Medial and lateral mitral annulus early diastolic (e’) velocities by tissue Doppler show a preserved longitudinal function and reversal of the usual relationship (so-called annulus reversus). (C) Thickened pericardium (arrow).

Twenty months after HTx, he presented to another center with progressive exertional dyspnea and lower extremity edema of one-week evolution. On exam, the patient was afebrile, normotensive, and tachycardic, and oxygen saturation was 96% (room air). Physical examination was notable for right-sided failure (marked elevation in jugular venous pressure, abdominal distention, and lower extremity edema). Initial laboratory analysis was within normal limits. Electrocardiogram was normal. An echocardiogram showed a thickened pericardium, septal bounce, and respiratory variation with a decrease in peak mitral E-wave velocity by >25% with short deceleration time. Medial e’ greater than lateral mitral annulus e’ velocity (annulus reversus), and a new deterioration in left ventricular function estimated at 45% (Figure 1A, B, C). Constrictive pericarditis was initially suspected, and he was referred to our center for evaluation by the heart transplant unit.

On direct questioning, he confirmed that he had recently discontinued immunosuppression. Admission blood levels of tacrolimus were <1 ng/mL. An urgent EMB and a right catheterization were performed. The pressures did not show a dip-and-plateau pattern. 

**Figure 2:**
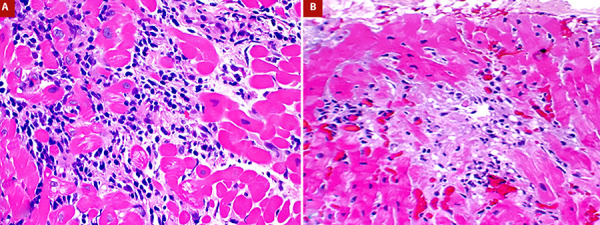
(A) Endomyocardial biopsy with grade 3R acute cellular rejection (Hematoxylin-eosin). (B) Endomyocardial biopsy with resolution of rejection.

Considering, the new ventricular dysfunction and the history of immunosuppressive therapy discontinuation, the most likely diagnosis was acute cellular rejection. Therefore, immunosuppressive medication was restarted, and he received an empiric intravenous pulse of methylprednisolone. One day later, EMB showed acute cellular rejection with multifocal aggressive infiltrate and myocyte damage, grade 3R from the International Society for Heart and Lung Transplantation classification (Figure 2A). The tacrolimus dose was adjusted to achieve a level of 12 ng/ml, and mycophenolate mofetil was restarted at a dosage of 1000 mg every 12 hours.

A follow-up EMB after intravenous steroids showed cellular rejection in resolution and improvement of the function of the cardiac graft and resolution of echocardiographic signs of constriction (Figure 2B).

## Discussion

Constrictive pericarditis presents a significant challenge in diagnosis, resembling various medical conditions and creating a diagnostic puzzle ever since its initial depiction 300 years ago as “Concretio Cordis.” It has the potential to imitate restrictive cardiomyopathy, endomyocardial fibrosis, as well as chronic liver and renal diseases ^3^.

Constrictive pericarditis is a rare complication in HTx, with a reported incidence of 1.4-3.9% ^2^^,^^4^. A crucial aspect of the diagnostic process involves maintaining a high clinical suspicion in patients exhibiting signs and symptoms of right-sided heart failure, especially when these manifestations appear disproportionate to pulmonary or left-sided heart disease. The cornerstone of accurate diagnosis lies in a keen awareness of CP’s potential presentation.

Extensive case series have delved into the nuances of chronic constrictive pericarditis, providing valuable insights into its varied clinical profiles. However, within the specific context of cardiac transplantation, the available literature is characterized by smaller case series and isolated reports detailing the emergence of constriction post-transplantation ^5^^,^^6^. These instances underscore the unique challenges in recognizing and managing CP in the transplant setting, necessitating a nuanced understanding of its atypical presentations and their association with postoperative events.

In this study, we present a noteworthy case involving a heart transplant recipient who exhibited clinical and echocardiographic features that closely resembled CP, ultimately leading to the diagnosis of acute allograft rejection. The complexity of distinguishing CP from other conditions manifesting as congestive heart failure in heart transplant recipients cannot be understated. To date, the literature is sparse on cases akin to ours, with only one previously reported instance of chronic cardiac rejection masquerading as constrictive pericarditis ^4^.

The diagnostic conundrum lies in the challenge of discriminating constrictive pericarditis from various other conditions that can precipitate symptoms of congestive heart failure in heart transplant recipients ^5^. This intricacy underscores the need for heightened clinical vigilance and meticulous diagnostic evaluation.

Right heart failure with pericardial effusion may mimic effusive-constrictive pericarditis, and it can be difficult to differentiate between them. The diagnosis of effusive-constrictive pericarditis should be based on hemodynamic findings, cardiac computed tomography, or CMR and confirmed by performing a combined pericardiocentesis and cardiac catheterization ^7^. Notably, the overlap in clinical presentation between CP and allograft rejection adds an additional layer of complexity to an already intricate scenario.

In our case, despite exhibiting echocardiographic signs suggestive of PC, such as pericardial thickening, respiratory variation, and annulus reversus, the crucial element in the diagnostic process was the patient’s medical history. The identification of irregular medication adherence and the onset of new ventricular dysfunction played a pivotal role in raising suspicion for acute rejection. This prompted an immediate catheterization, which did not reveal hemodynamics consistent with constrictive pericarditis. Subsequent pathology samples were obtained to further investigate the anatomical findings, ultimately confirming the presence of acute rejection.

It is crucial for healthcare providers to be cognizant of the potential diagnostic pitfalls associated with evaluating a physiology mimicking constrictive pericarditis. Our findings highlight the significance of performing a comprehensive and nuanced differential diagnosis, particularly focusing on the possibility of cellular rejection. The therapeutic implications of accurately identifying the underlying pathology cannot be overstated, emphasizing the importance of prompt and precise diagnosis in the management of heart transplant recipients.

Furthermore, caregivers and medical professionals should be attuned to the emergence of heart failure symptoms with unclear etiology in allograft recipients. An initiative-taking approach in considering a broad spectrum of potential entities in the differential diagnosis is imperative, ensuring a thorough and efficient evaluation process. By addressing these challenges head-on, we can enhance our ability to provide timely and targeted interventions, ultimately optimizing the care and outcomes for heart transplant recipients facing diagnostic uncertainties.
